# Liver DE(HP)toxification: luteolin as “phthalates-cleaner” to protect from environmental pollution

**DOI:** 10.1038/s44321-024-00158-3

**Published:** 2024-10-29

**Authors:** Federica Cappelli, Alessandro Mengozzi

**Affiliations:** 1 Department of Clinical and Experimental Medicine, University of Pisa, Pisa, Italy; 2 Center for Translational and Experimental Cardiology (CTEC), Department of Cardiology, University Hospital Zurich, University of Zurich, Schlieren, Switzerland

**Keywords:** Digestive System, Evolution & Ecology, Pharmacology & Drug Discovery

## Abstract

A Mengozzi and F Cappelli discuss a potential pharmacological strategy for phthalate detoxification of the liver as reported by S Chen, C Liu, W Zhang and colleagues, in this issue of *EMBO Mol Med*.

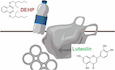

## Teaser

Environmental pollution is a major health problem that affects the health of individuals worldwide and is cumulative with other environmental stressors, contributing to the increasing prevalence of cancer, cardiometabolic, neurological and autoimmune diseases (Favé et al, 2018). Although being extensively studied, therapeutic strategies still rely primarily on exposure prevention, often with no option available to allow pollutant detoxification. In this issue of *EMBO Molecular Medicine*, Wang et al (Wang et al, 2024) investigated potential pharmacological strategies to allow the direct removal of phthalates, major environmental pollutants, from the liver.

Phthalates are chemical compounds widely used in various sectors of the plastics industry, from automotive, cosmetics, medications and personal care products to food packaging, including plastic bottles. Due to their intensive use, they are constantly being released into the environment and are considered to be environmental pollutants worldwide (Tuan Tran et al, 2022). Di-(2-ethylhexyl) phthalate (DEHP), in particular, has a chemical structure characterized by the absence of a chemical bond with polymers and is easily released from plastics and absorbed by the human body through several different but potentially synergistic routes: intestinal, transdermal and respiratory (Zhang et al, 2022). Once in the bloodstream, DEHP is rapidly distributed to tissues. In particular, DEHP accumulates in the liver, where it is converted to its active metabolites MEHP, MEOHP, and MEHHP.

Several studies have linked human exposure to phthalates to cardiometabolic damage. DEHP and its active metabolites act as endocrine disruptors and interfere with several pathways involved in deleterious lipid transport, oxidative stress, and subclinical inflammation. International biomonitoring studies have reported higher levels of DEHP in the population and numerous epidemiologic associations with cardiometabolic diseases (Gerofke et al, 2024; Wang et al, 2019; Mengozzi et al, 2019). In line with this, the detrimental relevance of phthalate exposure has been recognized by major regulatory agencies such as EPA/FDA (https://www.epa.gov/assessing-and-managing-chemicals-under-tsca/phthalates, https://www.fda.gov/food/food-additives-and-gras-ingredients-information-consumers/phthalates-food-packaging-and-food-contact-applications) and ECHA (https://www.foodpackagingforum.org/news/echa-regulation-updates-limits-of-dehp-in-fcms), which are continuously lowering the maximum allowable DEHP content in plastic articles. At present, however, only preventive strategies are available. Anti-inflammatory compounds could be supportive in reducing the effects of phthalate exposure, but drugs that can directly target phthalates to accelerate their elimination are currently unavailable and few studies have begun to explore this uncharted field (Mengozzi et al, 2021).

Wang et al started with a comprehensive network pharmacology analysis focusing on monomeric drugs from traditional Chinese medicine. From 128 detoxification formulas, they narrowed it down to 7 monomers and then tested their ability to remove DEHP. To do this, they developed a specific method to detect DEHP using a single-strand DNA-aptamer-based approach labeled with a Cy5 fluorescent probe. DNA-aptamer-based tracking systems are innovative, highly specific, with excellent tissue penetration and remarkable in vivo safety profile, making them ideal for tracking a small and lipophilic molecule such as DEHP (Lam et al, 2022). After setting up the method, they compared the efficacy of the 7 monomers on primary mouse hepatocytes exposed to high concentrations of DEHP and narrowed down their investigation to the most effective of the compounds, luteolin. They then tested the efficacy of a 14-day treatment with luteolin in an in vivo model of high chronic phthalate exposure, i.e., male C57BL/6J mice receiving 10 mg/kg/day DEHP for 28 days. The authors found that luteolin exerts a beneficial effect against DEHP exposure via hepatic detoxification, showing a reduction of DEHP (and related metabolites) levels in several organs, including kidney and adipose tissue. Luteolin also reduced DEHP-induced liver enzymes’ alterations and liver macrophages’ infiltration. Notably, luteolin treatment was also associated with a renoprotective effect, particularly relevant in this context as the kidney is, together with the liver, responsible for phthalates elimination from the body.

**Figure 1:**
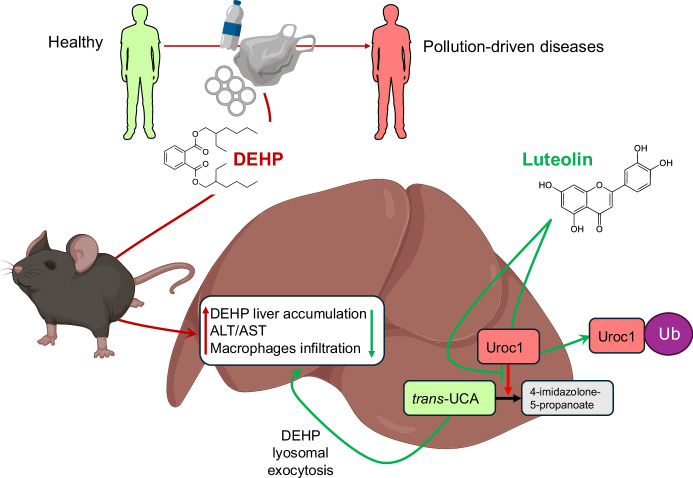
Luteolin reduces DEHP accumulation in the liver via the Uroc1/*trans*-UCA pathway. Phthalates, and in particular DEHP, are environmental pollutants widely used in the plastics industry, and chronic exposure to them promotes the development of environmental diseases such as cancer, neurological, cardiometabolic and autoimmune diseases. Currently, there is no established interventional strategy to remove DEHP from the body. In the study by Wang et al, the authors identified luteolin as a potential therapeutic strategy in this setting. In the mouse liver, luteolin promotes Uroc1 degradation via ubiquitination (Ub), thereby inhibiting the downstream conversion of *trans*-UCA to 4-imidazolone-5-propanoate. *trans*-UCA then acts as a functional metabolite that promotes DEHP lysosomal exocytosis, thereby removing the toxic compound from the liver and the associated liver injury. Although human studies are lacking, the authors’ work paves the way for therapeutic strategies that promote the removal of DEHP from the human body. Figure created with biorender.

The mechanism by which this beneficial effect of luteolin is achieved is an increased protein degradation of hepatic Uroc1, inhibiting *trans*-UCA transformation to 4-imidazolone-5-propanoate. In turn, higher levels of *trans*-UCA promotes lysosomal exocytosis of DEHP, facilitating the elimination of the toxic compound. Luteolin is a compound already known for its anti-inflammatory and antioxidant properties (Yao et al, 2023). However, these two effects were minor in the case of DEHP detoxification. Nevertheless, the combined effects of distinct luteolin properties may be even more relevant in the context of phthalates and may also be partly responsible for the renoprotective effect observed by the authors. Finally, by identifying *trans*-UCA as a downstream target of luteolin-mediated phthalate removal and demonstrating its role as a functional metabolite mimicking luteolin itself, they provide another potential target for novel pharmacological approaches (Fig. 1).

In conclusion, the study by Wang et al, opens a new perspective in the difficult challenge of tackling the effects of environmental pollution by identifying luteolin and its endogenous metabolite *trans*-UCA as compounds with therapeutic potential for phthalate removal. Although human clinical translation remains to be observed, the rigorous and innovative approach adopted by the authors inspires confidence and calls for immediate human observations, paving the way for the treatment of pollution exposure via removal of the toxic agent to prevent pollution-related disorders.
